# Effects of Graphic Health Warning on Tobacco Packs: A Cross-Sectional Study among Low Socioeconomic Group in Bangladesh

**DOI:** 10.1155/2021/1354885

**Published:** 2021-12-13

**Authors:** Md. Tuhin Mia, Mohammad Mahbub Alam Talukder, Md. Mokshead Ali, Md. Ismael

**Affiliations:** ^1^Institute of Social Welfare and Research, University of Dhaka, Dhaka 1000, Bangladesh; ^2^Accident Research Institute (ARI), Bangladesh University of Engineering and Technology, Dhaka 1000, Bangladesh; ^3^Consult-Aid Bangladesh (CAB), Mirpur, Dhaka 1213, Bangladesh; ^4^Institute of Education and Research, University of Dhaka, Dhaka 1000, Bangladesh

## Abstract

**Background:**

Tobacco use is a significant health concern in Southeast Asia, particularly in Bangladesh, where the greatest incidence of tobacco consumption occurs in a number of forms smoking, smokeless, and indigenous. The WHO Framework Convention on Tobacco Control (FCTC) requires tobacco product packaging to include adequate health warnings (text and visual). The study's objective is to investigate the effects of graphic health warnings on tobacco packs among Bangladeshi low socioeconomic groups. *Study Design*. Cross-sectional study.

**Methods:**

The study was conducted with 400 participants (low socioeconomic people) by using the systematic sampling technique through a semistructured questionnaire in Demra and Tongi industrial areas of Dhaka city in Bangladesh during September 2019-November 2020. Descriptive statistics (frequencies, percentages, means, and standard deviations) and inferential analysis (i.e., chi-square tests) were performed by Statistical Package for Social Sciences (SPSS version 25.0) to explore the relationship between the graphic warning and the use of tobacco.

**Results:**

This study illustrates that 89% of respondents smoke only cigarette or *bidi*, where 95.1% were daily smokers. About 72.2% reported pictorial warning message was more understandable while 90.8% reported the existing text warnings explicitly visualize the health harms. It has been found that there was a significant association between the respondent's opinion on the text warning that encouraged the respondent to quit tobacco use and the text messages “smoking causes throat and lung cancer” (*p* < 0.001) and “smoking causes respiratory problems” (*p* < 0.001). Around 96.7% knew about the graphic health warnings on the cigarette packets where 99.2% reported graphic warning explicitly visualizes the health harms. In graphical warnings, text messages have a great influence on quitting smoking where “smoking causes throat and lung cancer” (*p* < 0.001) and “smoking causes stroke” (*p* < 0.001). Nearly 79.2% of respondents thought the color of the graphic warning should be “Red” and a significant association between the color and the education level of the respondents explored here (*p* < 0.05).

**Conclusions:**

GHWs are more understandable on tobacco packets, and it has significant impacts on being aware of health consequences from tobacco consumption.

## 1. Introduction

Tobacco use is a significant health hazard in Southeast Asia. The region is a major producer of tobacco and tobacco-related goods. The region also has a high rate of tobacco use. It is consumed in a number of ways, including smoking, smokeless use, and indigenous consumption. Article 11 of the WHO Framework Convention on Tobacco Control (FCTC) requires nations to take effective measures to ensure that tobacco product packaging contains adequate health warnings [1]. Much of the evidence for the efficacy of graphic health warning labels on cigarette packets comes from research performed on well-educated people in Western countries [2]. The little data available indicates that health warnings have a comparable impact in underdeveloped nations [3]. The graphic health warnings are intended to provide a cost-effective method of raising public awareness about the risks of tobacco usage. At the moment, 42 nations representing about 42% of the world's population have made graphic health warnings obligatory on cigarette packaging [4]. The findings show that women, smokers, and those with lower levels of education considered the warning prototypes to be more negative than those with greater levels of education [5]. Health warning labels, particularly those with visual features, pose a challenge to the tobacco business since they are an inexpensive and effective method of smoking reduction. The tobacco industry has been aware of the efficacy of graphic health warning labels (GWHL) in Iceland since 1985 and has been successful in postponing GHWLs worldwide for over a decade [6]. However, since 2001, at least 63 nations have implemented combined health warnings [7].

Unlike many other consumer goods, cigarette packets are visible during use and are often kept in public view between uses. Cigarette packaging also functions as a vital connection to other types of tobacco promotion [8]. Package designs contribute to the reinforcement of brand images conveyed via other media and are critical in point-of-purchase marketing [9]. In Bangladesh, the requirements of the National Tobacco Control Act regarding health warnings on cigarette packets have been effectively enforced. In Bangladesh, both smoking and smokeless tobacco products are extensively utilized. The high frequency of smokeless tobacco use in the nation indicates a high degree of societal acceptance [10]. Tobacco products must have health warnings because they are extremely addictive and kill about half of their long-term users [11]. It is clear that text-only warnings are less successful than all visual forms, and graphic warnings get higher efficacy ratings than symbolic or testimonial warnings [12]. Graphic warnings create repulsive effect toward smoking and persuade smokers with those responses to think refraining from that. Nonetheless, in Bangladesh, graphical images on the tobacco packets have recently been implemented to discourage the smokers psychologically to quit smoking and encourage the nonsmoker not to start the habit of smoking. In early months of this year, first graphical images have been inserted in the cigarette packages and subsequently changing the graphic pictures in altering tobacco consumers more [13]. Thus, the study has been undertaken to explore the relationship between the graphic warning and the use of tobacco.

Therefore, this study will be a lighthouse towards the gray area from where the policy makers may have paradigm-shift-thought to impose strict rules for banning the production, sale, and use of tobacco. There is scarcity of information on the knowledge, attitude, and practice of graphical health warnings among low socioeconomic people of Bangladesh about pictorial warning on tobacco packs. This research intends to reduce a knowledge gap and provide new information on the effect of health warnings on smokers via the use of graphic warnings. The results will contribute to the increasing body of knowledge on health warnings and offer strong evidence for the efficacy of countries cigarette labeling policies, particularly for the country's low socioeconomic population.

## 2. Methods

### 2.1. Study Design, Duration and Area

**Figure 1:**
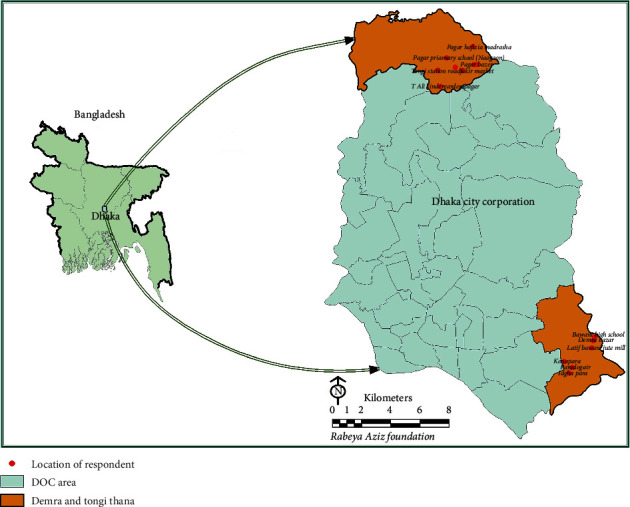
Geographical location of the study area.

This cross-sectional survey was conducted among low socioeconomic people who living in Demra and Tongi industrial areas under Dhaka district of Bangladesh during September 2019-November 2020. Geographical location of the study area is presented in Figure 1 [14].

### 2.2. Study Population

This systematic random sampling study targeted all the low socioeconomic people who live in Demra and Tongi industrial areas in Bangladesh to get a comprehensive picture of graphic warning of tobacco smoking among low socioeconomic populations.

### 2.3. Sample Size and Sampling Technique

The sample size of the study has been calculated by using the formula as follows [15]:

(1) $$\begin{array}{c} n=Z^{2}pq/d^{2}={\left(1.96\right)}^{2}\left(.5\right)\left(.5\right)/{\left(.05\right)}^{2}=384, \end{array}$$

where *n* is the desired sample size, *z* is the standard normal deviate (1.96 at 95% level of confidence), *p* is the prevalence of graphic health warnings (50% unknown prevalence), *q* = 1 − *p*, and *d* is the degree of accuracy required (5%).

Using systematic random sampling, the total of 400 respondents were selected by taking 50% prevalence and by adding 5% nonrespondent error.

Available literature could not give exact number of households in the study area. So, a sampling frame was developed through enumeration survey in the target area which is figure out in Table 1. From Tongi (6) and Demra (6), a total of 12 areas was selected purposively. In Tongi, there were about 2107 households, and in Demra, there are about 2033 households. A stratified proportionate sampling technique was used across those areas. In this research distributed, 400 (from Tongi 200 and Demra 200) samples according to the number of households of each area are shown in the following table. Based on the area household list, systematic random sampling technique was followed to select sample respondents from each area.

### 2.4. Data Collection Tools/Questionnaire

Systematic sampling was used to collect data, which were gathered through a semistructured questionnaire. The research gathered only quantitative data through face-to-face interviews. The printed version of the interview protocol with Bengali language was provided to and filled up by the data collectors because Bangladeshi people are native in Bengali language. The protocol was incorporated with different predetermined statements about people's knowledge, perceptions, attitudes, and practices towards graphic health warning on tobacco packs with yes, no, and remark options that must observe. Sociodemographic information was collected, including age, gender, education, marital status, educational qualification, occupation, and family income. To assess the status (knowledge, attitude, and practice of the respondents) of graphic health warning on tobacco packs, a total of 36 questions were included.

### 2.5. Statistical Analysis

The collected data from interviews were checked, cleaned, processed, and codified for ensuring reliability and validity of the data. Statistical significance was set at *p* < 0.05. All *p* values presented are two-tailed. The available latest version of Statistical Package for Social Sciences (SPSS version 25.0) and MS Excel was used to describe the basic features of the data in the study through frequencies and percentage. Descriptive statistics (frequencies, percentages, means, and standard deviations) and inferential analysis (i.e., chi-square tests) were performed to depict the status of graphic health warning on tobacco packs by measuring associations between independent (the graphic warning) and dependent variables (use of tobacco).

### 2.6. Ethical Approval

The research protocol was accepted by the BMRC's Dhaka Ethical Review Committee prior to the start of the project. Prior to performing the interview, all respondents verbally consented. A consent document was read to the respondent prior to the interview, and the interview began upon receipt of his/her consent. The study's freedom to deny and withdraw at any point was acknowledged. The respondents' details were kept strictly secret (approval number: BMRC/RP/Revenue/2019-20/607(6-98), date: 29/06/2020).

## 3. Results

### 3.1. Socioeconomic Background of the Respondents

It is evident from the Table 2 that the mean (±SD) age of the respondents was 38.4 (*±*12.4) years. The distribution of gender showed that less than one-fifteenth of the respondents (6.5%) were female. About one-fourth of the respondents (22.8%) did not have any formal education and slightly higher than one-fifteenth of the respondents (6.8%) had SSC or above level education. The highest majority of the respondents (91.7%) were married, and more than three-fourths of the respondents (78.5%) belonged to the nuclear family. The data also show the number of the family members of the respondents in two categories adult family members and child family members. In addition, the average number of adult family members was found as 3.26 and 1.41, respectively. Nearly one-sixth of the respondents (15.2%) had no child. Almost half of the respondents' (50.3%) monthly family incomes are Taka 10,001 to 15,000, followed by Taka 5,001-10,000 (29.5%). The data also show that about two-fifths of the respondents (40.3%) were service holders followed by 25.5% small businessmen and 24.5% day labor, of course, 5% was housewives having no income.

### 3.2. Usage of Tobacco, Knowledge, Perceptions, and Contemplation on Health Warning of the Tobacco Packet

Table 3 demonstrates that slightly below than nine-tenths (89%) of the respondents smoke only cigarette or *bidi,* and among the smokers, almost all the respondents (95.1%) were daily smokers. The mean age of smoking initiation was as 20.16 (±6.05) years. Among the SLT users about nine-tenths (88.6%) were daily users and a quarter of them practicing for 21 years or more. More than nine-tenths (93.8%) of the respondents knew that there was a health warning message on the cigarette pack. Only about three percent of them (2.9%) found cigarette packets where the type of warning message was text warning. Little higher than forty percent (40.5%) noticed that cigarette packets with pictorial type of warning messages during use and more than half of the respondents (56.5%) noticed cigarette packets both types of warning messages. Nearly three-quarter of the respondents (72.2%) reported pictorial warning message was more understandable, while a little higher than a quarter of respondents (26.4%) told that both type, of warning messages were easier to understand. The highest majority of the respondents (90.8%) thought that the existing text warnings explicitly visualize the health harms and (94.1%) knew that the text warning could create awareness about the health harms.

### 3.3. Association between Text Warning on the Tobacco Packet That Made the Respondents Concerned and Text Warning That Encouraged to Quit Tobacco Use

Table 4 illustrates that the association of the text warning on the tobacco packet that made the respondents concerned/worried and the text warning that encouraged the respondent to quit tobacco use. It has been found that there was significant association between the respondent's opinion on the text warning that encouraged the respondent to quit tobacco use and the text messages ‘smoking causes throat and lung cancer' (*p* < 0.001), ‘smoking causes respiratory problems' (*p* < 0.001), ‘smoking causes stroke' (*p* < 0.01), and ‘consumption of tobacco products causes mouth and throat cancer' (*p* < 0.05) made the respondents concerned.

### 3.4. Knowledge, Perceptions, and Contemplation on the GHW of the Cigarette/*Bidi* Packet

Table 5 describes that almost all of the respondents (96.7%) knew about the graphic health warnings on the cigarette packets. Hundred percent of respondents noticed graphic warnings on cigarette/bidi pack are clearly visualized in Bangladesh. Almost the same percentage of the respondents (96.4%) saw “ulcer on throat” and 3.6% of them noticed “cancer on mouth” as the graphic warning. About 84.8% of the respondents noticed the text message on the newly introduced graphic warning packs and only 36.7% of the respondent could read clearly. The highest majority (88.6%) of the respondents gave first look into the pictorial health warning on packs. Almost all of the respondents (99.2%) reported graphic warning explicitly visualizes the health harms; and the graphic warnings made them aware of the health harms. It also shows that almost all of the respondents (99.7%) were concerned of the graphic warning. About half of the respondents (49.2%) were aware regarding the health risk of smoking. The data also represent that almost all the respondents (97.7%) observed graphic warning to quit tobacco using, and nearly, seventy percent (69.0%) of them think that the graphic warnings will prohibit starting smoking by the young people.

### 3.5. Association between the Most Effective GHW and Quitting Smoking by Younger People

Table 6 shows that the associations of new warning labels will make young people less likely to start smoking by the opinions on the most effective GHW likely to quit smoking. The findings showed that the associations were significant. They are ‘smoking causes throat and lung cancer' (*p* < 0.001), ‘smoking causes respiratory problems' (*p* < 0.001), ‘smoking causes stroke' (*p* < 0.001), ‘smoking causes heart disease' (*p* < 0.05), and ‘consumption of tobacco products causes mouth and throat cancer' (*p* < 0.01).

### 3.6. Respondents' Recommendation on Cover, Color, and Design of GHW of the Cigarette

Table 7 shows that 83.0% of the respondents thought that the graphic warnings should cover ‘100% of the cigarette packet.' More than three-fourths (79.2%) of the respondents thought that the color of the graphic warning should be ‘Red.' More than half (54.8%) of them thought that the design of GHW should be larger and 44.5% of them thought that the font size of text message of the GHW should be bigger.

### 3.7. Association of Respondents' Recommendation on Cover, Color, and Design of GHW by Education Level

The findings in Table 8 show the remarks of the association respondents' recommendations on the cover, color, and design of GHW by education levels. Among the respondents who choose Red color as more attractive color of the graphic warning, nearly one-fifth of the respondents (19.6%) were illiterate, about 40% of the respondents (40.1%) had primary education. 32.8% of them had secondary level education, and 7.6% of them was at of SSC level and above. This is a significant association of the color and the education level of the respondents (*p* < 0.05).

## 4. Discussion

Age, sex, and education all have a significant role on the socioeconomic and demographic characteristics of respondents about their usage of tobacco products. These three factors are important factors that affect health and the characteristics of an individual. The findings of this study reveal that the mean age of the respondents was 38.4 (SD ± 12.40) years. It is comparatively similar to other study where more than half of the Bangladeshi people are over the age of 25 years smoke tobacco [16]. In the gender distribution, this study shows a very few percent of the participants were found to be female who have smoking habit. Another study reported dissimilar findings where in Bangladesh, smokeless tobacco use among the females (28.0%) is comparable to the males (26%) [17]. In this study, it was seen that most of the respondents used to smoke only cigarette or *bidi*, where a very few about 9.0% of them were using smokeless tobacco, and the rest of the participants were using both. These results corroborated the study's conclusions that 33% of men and 18% of females use smokeless tobacco in India; approximately 85.0% of female users solely use smokeless tobacco [17]. This study explored that most of the study respondents noticed the health warning on tobacco packets as a text form, and many of them noticed recently implemented pictorial warning in Bangladesh. It was shown in certain research that the effectiveness of text-only warnings was diminished when compared to visual approaches. Symbolic and testimonial warnings were given lower efficacy ratings than graphic warnings [12].

The present study discovered that only few percent of respondents found cigarette packets where the type of warning message was text warning. Little higher than forty percent noticed that cigarette packets with pictorial type of warning messages during use and more than half of the respondents noticed cigarette packets both types of warning messages. This contradicts results from previous studies where it was discovered that almost all of the respondents observed GHW and that it was very constant across different groups. It is also seen from another previous study that 88.4% students, 86.9% job holders, and 79.4% of day laborers noticed both the pictorial and text warning [18]. This study explored that nearly most of the respondents reported pictorial warning message was more understandable while a little higher than a quarter of respondents told that both type, of warning messages were easier to understand. It is a little bit inconsistent with other existing study findings where it shows most of the respondents who felt the GHWs were sending out a clear message were day laborers (93.2%) and most of the respondents who disagreed were students (74.4%) [18]. This study has shown that one of the most influential sources of health information for the public is the warnings on cigarette packaging. Approximately 75.2% of respondents agreed that warning labels on cigarette packs encourages smokers to stop. This is also consistent with an earlier finding, which showed that the images that feature graphic, fearful depictions of smoking may help to increase motivation to quit, since they increase the motivation to engage in behavior to prevent health problems, and increase the motivation to give up smoking [7]. The present study noticed that almost all of the respondents knew about the graphic health warnings on the cigarette packets, which were recently included in the tobacco packs of Bangladesh, particularly in the cigarette packs. Almost all of the respondents who knew mentioned that they saw “ulcer on the throat” and “cancer on the mouth,” as the graphic warnings in recent time either in the cigarette packs or through mass media, which was effective and understandable. This is consistent with the findings of another study; the majority of the participants (72.7%) were aware of statutory and pictorial warnings present on the cigarette packs. About 69.6% of them said they could understand pictorial warnings given on the cigarette packs, and 50.8% of them said pictorial warning on tobacco products encouraged them to quit tobacco habits [19]. As this study results revealed that recently implemented graphic health warning giving strong messages to stop cigarette initiation compared to text messages, particularly among young and low educated people. The similar results were documented in another study that picture health warnings provide evidence to suggest that images are more effective than text in promoting smoking cessation, as well as increasing public health awareness and perceptions of risk [20].

This study presented that almost all of the respondents were aware of the existing graphic warnings explicitly visualizing the health harms and the graphic warning made them aware of the health harms. That can be comparable to the study outcomes where it was indicated that why 42 countries were interested. It also shows that why approximately 42% of world population making graphic health warnings mandatory on cigarette packages [4]. In this study, the majority of the respondents reported that the graphic warning should cover ‘100% of the cigarette packets,' which would be most effective and the people would be more alert on the consequences of tobacco uses through visualizing larger GHW. On the other hand, big detailed graphic health warnings have a significant effect on teenage smokers, causing them to significantly reduce their use. These findings corroborate another research concluded that bigger, graphic health warnings are more effective than the text-only warnings presently in use. At least 63 nations worldwide have implemented combined health warnings since 2001 [7]. Another research established that visual warnings should always be more apparent and acceptable than text-only warnings [12]. A present study illustrated that hundred percent respondents noticed graphic warnings on cigarette/bidi pack in Bangladesh. In this regard, this finding is slightly inconsistent with another study where although almost 80% of all tobacco packages and containers included some kind of GHW, substantial loopholes existed to reach 100% compliance [13].

## 5. Conclusions

Smoking has a serious health risks, which causes countless negative impacts on health. People consume tobacco in varieties of form-smoking, smokeless, etc. People were aware that smoking is very dangerous to their life. However, most of the people were using tobacco daily; smoking became a part of their routine work. Warning labels have impacts on changing behavior of the tobacco users. Those who were aware of warning signals, the majority of them expressed intention either to reduce the quantity of smoking or quit tobacco due to health hazards. The study found a favorable attitude among the majority of the respondents regarding the recent introduction of the pictorial warnings in Bangladesh, which had to be enlarged. GHWs are more comprehensible than other warnings on tobacco packets, and health warnings on tobacco packets greatly influence the awareness of smoking's health effects. All of these factors influenced respondent's intention to stop smoking. Thus, graphic health warnings aid in the reduction of tobacco use in the nation, which in turn contributes to the long-term reduction of tobacco-related illness and death, thus enhancing Bangladesh's public health.

## Figures and Tables

**Table 1:** Sample distribution across the study areas.

Demra	No. of HH	Proporti on	Sample size	Tongi	No. of HH	Proporti on	Sample size
Bawani High School	263	0.129	26	Basic Fakir, Market	244	0.116	23
Demra Bazar	432	0.213	42	Pagar Bazer	180	0.0854	17
Eidgah Para	322	0.158	32	Pagar Hafezia Madrasa	298	0.1414	28
Kona Para	326	0.160	32	T Ali KG School	269	0.1277	26
Latif Bawani Jute Mills	526	0.259	52	Station Road	792	0.3759	75
Para Dogair	164	0.081	16	Pagar primary school area	324	0.1538	31
	2033	1.000	200		2107	1.000	200

**Table 2:** Socioeconomic background of the respondents.

Variable	Number (*N* = 400)	Percentage
Mean age ± SD	38.4 ± 12.4	
Age group		
20 ≤ 24	42	10.5
25-34	115	28.8
35-44	117	29.2
45-54	74	18.5
≥55	52	13.0
*Gender*		
Male	374	93.5
Female	26	6.5
*Education level*		
No formal education	91	22.8
Primary	160	40.0
Secondary	122	30.5
SSC & above	27	6.8
*Marital status*		
Unmarried	33	8.3
Married	367	91.7
*Family type*		
Nuclear	314	78.5
Joint	86	21.5
*Family members*		
Adults		
Mean ± SD	3.26 ± 1.26	
1-3	248	62.0
4-6	146	36.5
≥7	6	1.5
Child		
Mean ± SD	1.41 ± 0.9	
0	61	15.2
1-2	299	74.8
3-4	40	10.0
*Occupation*		
Housewife	20	5.0
Day labor	98	24.5
Service	161	40.3
Small business	102	25.5
Unemployed	9	2.3
Student	4	1.0
Others	6	1.5
*Family income (BDT)*		
≤5,000	24	6.0
5,001-10,000	118	29.5
10,001-15,000	201	50.3
15,000+	57	14.3

**Table 3:** Respondent's usage of tobacco, knowledge, perceptions, and contemplation on health warning of the tobacco packet.

Variables	Number (*N* = 400)	Percentage
*Type of tobacco use* (*N* = 400)		
Smoking	356	89.0
Smokeless (SLT)	36	9.0
Both	8	2.0
*Frequency of smoking tobacco* (*N* = 364)		
Daily	346	95.1
Occasionally	18	4.9
*Initiating age of smoking (in year)* (*N* = 364)		
Mean ± SD	20.2 ± 6.1	
≤17	112	30.8
18-24	171	47.0
25-31	67	18.4
≥32	14	3.8
*Frequency of using SLT* (*N* = 44)		
Daily	39	88.6
Occasionally	5	11.4
*Duration of using SLT (in year)* (*N* = 44)		
Mean ± SD	18.4 ± 9.4	
≤5	3	6.8
6-10	6	13.6
11-15	12	27.3
16-20	12	27.3
≥21	11	25.0
*Noticed health warning on the tobacco packet respondent's used* (*N* = 400)		
No	25	6.3
Yes	375	93.8
*Type of health warning noticed elsewhere* (*N* = 375)		
Text warning only	11	2.9
Pictorial warning only	152	40.5
Both	212	56.5
*The type of health warning more understandable to respondents* (*N* = 212)		
Text warning only	3	1.4
Pictorial warning only	153	72.2
Both	56	26.4
*Visualize the health harms on the text warning* (*N* = 371)		
No	34	9.2
Yes	337	90.8
*The text warning makes aware on the health harms* (*N* = 371)		
No	22	5.9
Yes	349	94.1

**Table 4:** Association between text warning on the tobacco packet that made the respondents concerned and text warning that encouraged to quit tobacco use.

Text warning message that made concerned/worried	Respondent's opinion on the encouragement to quit tobacco use		Chi-square	*p* value^∗^
	No *N* (%)	Yes *N* (%)		
*Smoking causes throat and lung cancer*				
No	3 (4.0)	72 (96.0)	10.63	<0.001
Yes	54 (19.7)	220 (80.3)		
*Smoking causes respiratory problems*				
No	37 (49.3)	38 (50.7)	29.07	<0.001
Yes	220 (80.3)	54 (19.7)		
*Smoking causes stroke*				
No	47 (62.7)	28 (37.3)	10.18	<0.01
Yes	220 (80.3)	54 (19.7)		
*Smoking causes heart disease*				
No	46 (61.3)	29 (38.7)	1.57	0.133
Yes	189 (69.0)	85 (31.0)		
*Consumption of tobacco products causes mouth and throat cancer*				
No	65 (86.7)	10 (13.3)	4.79	<0.05
Yes	258 (94.2)	16 (5.8)		

^∗^Pearson's chi-squared test.

**Table 5:** Knowledge, perceptions, and contemplation on the GHW of the cigarette/*bidi* packet.

Variable	Number (*N* = 400)	Percentage
*Knowledge of the respondent on graphic health warning on cigarette/bidi packs*		
No	13	3.3
Yes	387	96.7
*Noticed graphic warnings on cigarette/bidi pack in Bangladesh* (*N* = 387)		
Yes	387	100.0
*Type of graphic warning seen on cigarette/bidi in Bangladesh* (*N* = 387)		
Ulcer on the throat	373	96.4
Cancer on mouth	14	3.6
*Noticed text message in the new graphic warning* (*N* = 387)		
No	59	15.2
Yes	328	84.8
*Visibility of reading the text message on the graphic warning* (*N* = 387)		
Read clearly	142	36.7
Read little	134	34.6
No	55	14.2
Cannot read	56	14.5
*Noticed first on the graphic warning* (*N* = 387)		
Brand name	34	8.8
Pictorial health warning	343	88.6
Color and design of the packet	9	2.3
Text message	1	0.3
*Visualize the health harms on the graphic warning*		
No	3	0.8
Yes	384	99.2
*The graphic warning makes aware on the health harms*		
No	3	0.8
Yes	384	99.2
*Any graphic warning that makes worried*		
Yes	1	0.3
No	386	99.7
*Degree of worries about the health risk of smoking that are in the graphic warning*		
Not at all	2	0.5
A little bit	119	30.7
To some extent	190	49.1
A lot	76	19.6
*The graphic warning encourage the respondent to quit tobacco use*		
Yes	9	2.3
No	378	97.7
*The graphic warning will prohibit starting smoking by the young people*		
No	32	8.3
Yes	267	69.0
Do not know	88	22.7

**Table 6:** Association between the most effective GHW and quitting smoking by younger people.

Opinion on most effective GHW likely to quit smoking	Opinion on new warning labels less likely to start smoking by younger			Chi-square	*p* value^∗^
	No *N* (%)	Yes *N* (%)	Do not know *N* (%)		
*Smoking causes throat and lung cancer*					
No	8 (8.7)	84 (91.3)	81 (28.2)	19.761	<0.001
Yes	90 (32.3)	189 (67.7)	7 (7.0)		
*Smoking causes respiratory problems*					
No	45 (48.9)	47 (51.1)	48 (53.9)	59.693	<0.001
Yes	244 (87.5)	35 (12.5)	40 (13.4)		
*Smoking causes stroke*					
No	49 (53.3)	43 (46.7)	44 (38.3)	18.423	<0.001
Yes	214 (76.7)	65 (23.3)	44 (16.2)		
*Smoking causes heart disease*					
No	64 (69.6)	28 (30.4)	26 (31.0)	4.579	<0.05
Yes	224 (80.3)	55 (19.7)	62 (20.5)		
*Consumption of tobacco products causes mouth and throat cancer*					
No	72 (78.3)	20 (21.7)	21 (17.5)	6.584	<0.01
Yes	178 (63.8)	101 (36.2)	67 (25.1)		

^∗^Pearson's chi-squared test.

**Table 7:** Respondents' recommendation on cover, color, and design of GHW of the cigarette.

Variable	Number (*N* = 400)	Percentage
*Recommendation on the GHW covering of the cigarette packet*		
100% of the pack	332	83.0
50% of the pack	66	16.5
Do not know	2	0.5
*The color that more eye catching for the graphic warning*		
Red	317	79.2
Green	30	7.5
Black	1	0.2
Any color	35	8.8
Not sure	17	4.2
*The type of design of GHW on the cigarette packet*		
GHW should be larger	219	54.8
Bigger font size of the text message of GHW	178	44.5
Simple color needs to be used	3	0.8

**Table 8:** Association of respondents' recommendation on the cover, color, and design of GHW by education level.

Recommendation on the cover, color, and design of GHW	Respondent's education level				Chi-Square	*p* value^∗^
	Illiterate *N* (%)	Primary *N* (%)	Secondary *N* (%)	SSC+ *N* (%)		
*Color to be more eye catching*						
Red	62 (19.6)	127 (40.1)	104 (32.8)	24 (7.6)	10.60	<0.05
Any other color	25 (37.9	21 (31.8)	17 (25.8)	3 (4.5)		
*The type of design of GHW on cigarette pack*						
GHW should be larger	43 (19.6)	88 (40.2)	66 (30.1)	22 (10)	10.011	0.124
Bigger font size	47 (26.4)	71 (39.9)	55 (30.9)	5 (2.8)		
Simple color needs to be used	1 (33.3)	1 (33.3)	1 (33.3)	0 (0)		
*The cover page (%) should the warning*						
100% of the pack	79 (23.8)	132 (39.8)	104 (31.3)	17 (5.1)	9.716	<0.05
50% of the pack	11 (16.7)	27 (40.9)	18 (27.3)	10 (15.2)		

^∗^Pearson's chi-squared test.

## Data Availability

The authors confirm that all data underlying the findings are fully available without any restriction. All descriptively presented open access data are properly arranged by appropriate citations and referencing systems.
