# Corrigendum to “Effects of Graphic Health Warning on Tobacco Packs: A Cross-Sectional Study among Low Socioeconomic Group in Bangladesh”

**DOI:** 10.1155/2022/9804931

**Published:** 2022-07-18

**Authors:** Mohammad Mahbub Alam Talukder, Md. MoksheadAli, Md. Tuhin Mia, Md. Ismael

**Affiliations:** ^1^Accident Research Institute (ARI), Bangladesh University of Engineering and Technology, Dhaka 1000, Bangladesh; ^2^Consult-Aid Bangladesh (CAB), Mirpur, Dhaka 1213, Bangladesh; ^3^Institute of Social Welfare and Research, University of Dhaka, Dhaka 1000, Bangladesh; ^4^Institute of Education and Research, University of Dhaka, Dhaka 1000, Bangladesh

In the article titled “Effects of Graphic Health Warning on Tobacco Packs: A Cross-Sectional Study among Low Socioeconomic Group in Bangladesh” [1], there was an error in the order of the authors. Md. Tuhin Mia should have been listed as the third author in the author list.

Additionally, Dr. Md. Tuhin Mia was incorrectly listed as the corresponding author. The correct corresponding author is Prof. Mohammad Mahbub Alam Talukder.

This is corrected as shown above.
